# Steroid pretreatment of organ donors does not impact on early rejection and long‐term kidney allograft survival: Results from a multicenter randomized, controlled trial

**DOI:** 10.1111/ajt.15252

**Published:** 2019-02-01

**Authors:** Roman Reindl‐Schwaighofer, Alexander Kainz, Kira Jelencsics, Andreas Heinzel, Gabriela Berlakovich, Ádám Remport, Georg Heinze, Robert Langer, Rainer Oberbauer

**Affiliations:** ^1^ Department of Nephrology Medical University of Vienna Vienna Austria; ^2^ Department of Surgery Medical University of Vienna Vienna Austria; ^3^ Department of Nephrology Semmelweis University Budapest Hungary; ^4^ Center for Medical Statistics Informatics and Intelligent Systems (CEMSIIS) Medical University of Vienna Vienna Austria; ^5^ Department of Surgery Elisabethinen Krankenhaus Linz Austria

**Keywords:** clinical trial, critical care/intensive care management, donors and donation: deceased, graft survival, kidney transplantation/nephrology, organ procurement and allocation, translational research/science

## Abstract

Steroid pretreatment of deceased donors reduces inflammation in allografts and is recommended by organ procurement guidelines. The impact on long‐term graft outcome, however, remains elusive. In this multicenter randomized controlled trial, 306 deceased donors providing organs for 455 renal transplant recipients were randomized to 1000 mg of methylprednisolone or placebo prior to organ procurement (ISRCTN78828338). The incidence of biopsy‐confirmed rejection (Banff>1) at 3 months was 23 (10%) in the steroid group and 26 (12%) in the placebo group (*P* = .468). Five‐year functional graft survival was 84% and 82% for the steroid group and placebo group, respectively (*P*‐value = .941). The hazard ratio of functional graft loss was 0.90 (95% confidence interval 0.57‐1.42, *P* = .638) for steroid vs placebo in a multivariate Cox model. We did not observe effect modification by any of the predictors of graft survival and treatment modality. A robust sandwich estimate was used to account for paired grafts of some donors. The mean estimated GFR at 5 years was 47 mL/min per 1.73 m^2^ in the steroid group and 48 mL/min per 1.73 m^2^ in the placebo group (*P* = .756). We conclude that steroid pretreatment does not impact on long‐term graft survival. In a donor population with higher risk of delayed graft function, however, repetitive and higher doses of steroid treatment may result in different findings.

## Abstract 2

Steroid pretreatment of deceased organ donors using a single 1000 mg dose of methylprednisolone does not reduce the incidence of biopsy‐confirmed acute rejection episodes within the first 3 months posttransplantation and does not improve 5‐year functional graft survival compared to placebo, despite reduction of inflammation signatures in preimplantation biopsies.

**Abbreviations**
BCAR: biopsy‐confirmed acute rejection
GFR: glomerular filtration rate
KDPI: kidney donor risk index

## 1 INTRODUCTION

Brain death triggers a complex series of pathophysiological changes that drive alterations of gene expression in donor organs.^1^, ^2^, ^3^ Kidney allografts from brain‐dead donors are characterized by a pro‐inflammatory state when compared to live kidney donation, which correlates with the incidence and severity of acute kidney injury in the allograft.^4^, ^5^, ^6^, ^7^ Strategies to optimize and preserve quality and function of the allograft are needed.^8^ Anti‐inflammatory treatment of the donor prior to organ procurement provides a promising strategy to improve transplant outcome. Nonrandomized and retrospective studies from the late 1970s and early 1980s suggested that steroid pretreatment of donors may improve short‐ and long‐term graft survival.^9^, ^10^, ^11^

We previously reported the short‐term results of a randomized controlled trial on systemic steroid pretreatment of donors prior to organ retrieval.^12^ We showed that steroid pretreatment of donors effectively reduced the molecular inflammation signature in preimplantation transplant kidney biopsy specimens. However, there was no reduction in the incidence of delayed graft function after steroid pretreatment compared to placebo control.

Current organ procurement guidelines advocate steroid pretreatment of organ donors before organ procurement despite the low level of evidence.^13^ Long‐term effects of anti‐inflammatory treatment of the donor on kidney allograft and patient outcome remain elusive. We report here the long‐term outcome of the multicenter, randomized, controlled steroid pretreatment of organ donors trial.

## 2 MATERIALS AND METHODS

### 2.1 Study population

The study design and randomization of the multicenter study have been described previously.^12^ In brief, between February 2006 and November 2008, 306 deceased donors from 3 transplantation centers in Europe were randomly assigned to receive corticosteroids or placebo at least 3 hours before organ retrieval. Donors were enrolled by the transplant coordinator. Randomization was done in blocks by 4 and stratified by donor age using a threshold of 50 years. The blinded study drug or placebo was sent to the donor site by the transplant coordinator. No information on comedication during the donor management prior to study enrollment was available. A total of 455 kidney grafts were finally allocated to recipients who were transplanted at the participating study centers: 238 patients received an organ from a steroid‐pretreated donor and 217 patients received an organ from a donor treated with placebo. All kidneys were statically stored in cold preservation solution and none was machine perfused. Primary outcome was the rate of delayed graft function at 1‐week follow‐up.

Recipients received a perioperative steroid bolus of 40 mg of dexamethasone. Steroids were then tapered to a maintenance dose of 5 mg of prednisolone per day over the course of 3 months. Details on induction therapy are stated in Table 1. All patients were started on a calcineurin inhibitor–based immunosuppressive regimen.

**Table 1:** Demographics at time of transplantation for steroid treatment and placebo group

Recipients	n	Steroid (n = 228)	Placebo (n = 212)
Age (y)	440	50 ± 14	50 ± 14
Recipient sex	440		
Male	296	157 (68.9%)	139 (65.6%)
Female	144	71 (31.1%)	73 (34.4%)
Renal diagnosis	440		
Glomerulonephritis	114	60 (26.3%)	54 (25.5%)
Vascular	4	1 (0.4%)	3 (1.4%)
Diabetes	49	27 (11.8%)	22 (10.4%)
Other	273	140 (61.4%)	133 (62.7%)
Transplant number	440	1 (1, 1)	1 (1, 1)
Cold ischemic time (h)	440	16.7 ± 13.6	16.8 ± 15.3
Sum of HLA mismatches	440	3 (2, 4)	3 (2, 4)
Panel reactive antibodies (%)	440	0 (0, 2)	0 (0, 0)
Induction therapy	440		
None	257	134 (58.8%)	123 (58.0%)
Anti–CD25	165	82 (36.0%)	83 (39.2%)
ATG	18	12 (5.3%)	6 (2.8%)
Donors			
Donor age (y)	264	47 ± 15	49 ± 14
Donor sex	264		
Male	147	72 (54.1%)	75 (57.3%)
Female	117	61 (45.9%)	56 (42.7%)
Last creatinine of donor (mg/dL)	264	0.89 ± 0.31	0.89 ± 0.38
Donor cause of death	283^a^		
Traumatic	77	41 (30.8%)	36 (27.5%)
Intracranial hemorrhage	175	86 (64.7%)	89 (67.9%)
Cardiac arrest	9	5 (3.8%)	4 (3.1%)
Other	22	11 (8.3%)	11 (8.4%)
Vasopressors used	264		
Yes	231	111 (83.5%)	120 (91.6%)
No	33	22 (16.5%)	11 (8.4%)
Multiorgan donor	240		
Yes	69	30 (24.6%)	39 (33.1%)
No	171	92 (75.4%)	79 (66.9%)
Timing of study drug dosing and organ recovery	440		
3‐12 h	428	225 (99%)	203 (96%)
>12 h	12	3 (1%)	9 (4%)

a 19 donors were counted for 2 different causes.

To assess long‐term outcomes of steroid pretreatment, we prospectively followed the patients enrolled in the steroid pretreatment trial for 5 years. The CONSORT flow chart is provided in the supplemental data file (Figure S1).

The study protocol was approved by the institutional review board (Ethical Committee of the Medical University of Vienna, Vienna, Austria, EK‐067/2005) and the Eurotransplant kidney advisory committee (6021KAC06) at each study site. All clinical research has been performed in adherence to the Declaration of Helsinki. The clinical and research activities being reported are consistent with the Principles of the Declaration of Istanbul as outlined in the “Declaration of Istanbul on Organ Trafficking and Transplant Tourism.”

### 2.2 Outcomes

The co‐primary outcome of this study was incidence of biopsy‐confirmed acute rejection (BCAR) episodes within the first 3 months and death‐censored graft survival at 5 years. BCAR was defined as T cell–mediated rejection Banff ≥1 excluding borderline lesions.^14^ As secondary outcomes, we analyzed actual graft survival at 5 years as well as allograft function over time using the estimated glomerular filtration rate (eGFR) computed by the MDRD formula.^15^ Investigators analyzing the data were blinded for the study treatment.

### 2.3 Statistical analysis

Demographics data are reported as mean and standard deviation or median and first and third quartile for nonnormal distributed variables. In case of categorical variables, absolute count and percentage were computed.

The Kaplan‐Meier method was used to visualize graft survival and the log rank test was used for evaluating the differences between steroid and placebo treatment. A Cox proportional hazards model was used to evaluate graft loss. In addition, a clinical expertise model was calculated using donor age, donor sex, and sum of HLA mismatch as covariates. The proportional hazard was verified by Schoenfeld residuals. A robust sandwich estimate was used to account for nonindependence if both kidneys from a donor were assigned to patients in a participating study center. Interaction analysis of treatment assignment with predictors of functional graft loss was performed using age stratification and site of randomization as additional covariables. No correction for multiple testing was applied.

To estimate differences of eGFR trajectories between the treatment groups, we used a linear mixed model with a spatial covariance matrix, because measurements were obtained at 1, 3, and 6 months as well as every year after transplantation. The intercept and time variables were considered as a random effect. Patients who lost the graft were censored in the eGFR analyses.

The trial was powered to detect a 50% reduction in delayed graft function at 1 week.^12^ We calculated the detectable effect size for a difference in 5‐year graft survival between both groups, assuming a median graft survival of 10 years in our data set. This analysis showed that 220 recipients in each group would allow us to detect a hazard ratio (HR) <0.63 or >1.72 with power of 80% at an alpha of 0.05 (Figure S2).

For all analyses, SAS 9.4 for Windows (Cary, NC) was used. A *P*‐value <.05 was considered significant.

## 3 RESULTS

Follow‐up for 5 years was complete in 440 of 455 patients (97%). Ten patients in the steroid group and 5 in the placebo group were lost to follow‐up. Demographic data of transplant recipients and donor treatment interventions are listed in Table 1.

### 3.1 Biopsy‐confirmed acute rejection

The incidence of BCAR within 3 months was 10% (23/228) in the steroid treatment group and 12% (26/212) in the placebo group, showing no statistically significant difference (−2.2%, 95% confidence interval [CI] −7.9% to 3.5%, *P* = .468). Table S1 shows Banff classification scores for both groups. No antibody‐mediated rejection was observed.

### 3.2 Functional graft survival

**Figure 1:**
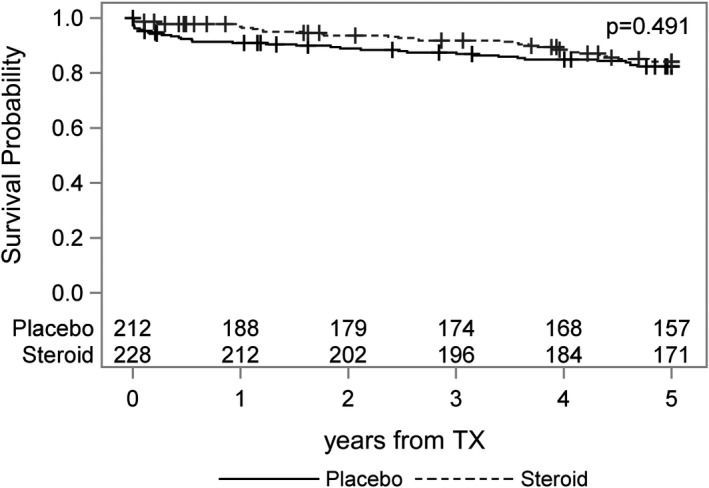
Functional graft survival (death censored). Numbers indicate the patients at risk. TX, transplant

A total of 70 graft losses were observed during the 5‐year follow‐up period, 34 in the steroid and 36 in the placebo group. Death‐censored graft survival at 5 years was 84% for the steroid treatment group and 82% for the control group, resulting in a difference of 2.1% (95% CI −4.8% to 9.1%, *P* = .617). The time to event analysis is visualized by a Kaplan‐Meier plot in Figure 1. The unadjusted hazard ratio of functional graft loss for steroid vs placebo was 0.85 (95% CI 0.53 to 1.35, *P* = .491). The risk of functional graft loss remained materially unchanged after adjustment for donor age and sex and HLA mismatch (HR 0.90, 95% CI 0.57 to 1.42, *P* = .638) (Table 2).

**Table 2:** Cox proportional hazards model for functional graft loss

Parameter	Hazard ratio	95% confidence limits		*P*‐value
Donor treatment (steroid vs placebo)	0.90	0.57	1.42	.638
Donor age (per y)	1.03	1.01	1.04	.003
Donor sex (female vs male)	1.07	0.67	1.72	.768
Sum HLA mismatch (per mismatch)	1.19	0.96	1.47	.105

There were also no statistically significant differences in actual graft survival, patient survival, and cumulative incidence of graft loss using death as a competing risk for functional graft loss between both groups (Figures S3‐S5).

### 3.3 Interaction analysis

**Figure 2:**
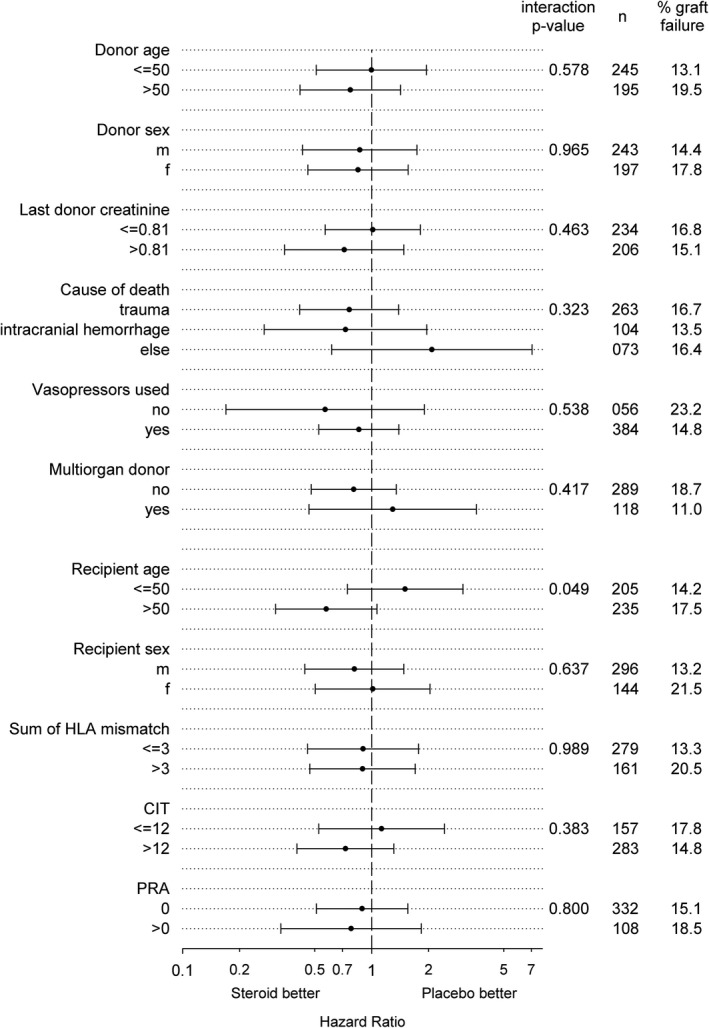
Interaction analysis of treatment assignment with predictors of functional graft loss. No effect modification was observed, *P*‐values were not corrected for multiple testing. CIT, cold ischemic time; PRA, panel reactive antibodies

Interaction analysis of treatment modality (steroid treatment vs placebo) with selected demographic variables on functional graft loss did not exhibit any effect modification (Figure 2). The only significant result was the interaction with older recipient age, pointing towards a potential benefit from steroid treatment in recipients older than 50 years (*P* = .049). However, no correction for multiple testing was performed as indicated above.

The Schoenfeld residuals for any model of the survival analysis did not show any deviation from the proportional hazards assumption.

### 3.4 Transplant function

**Figure 3:**
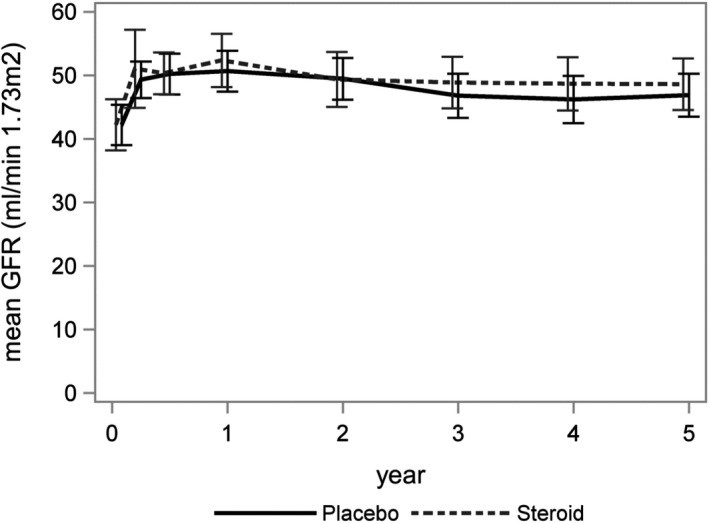
Trajectories of mean estimated GFR with 95% confidence intervals. GFR, glomerular filtration rate

**Figure 4:**
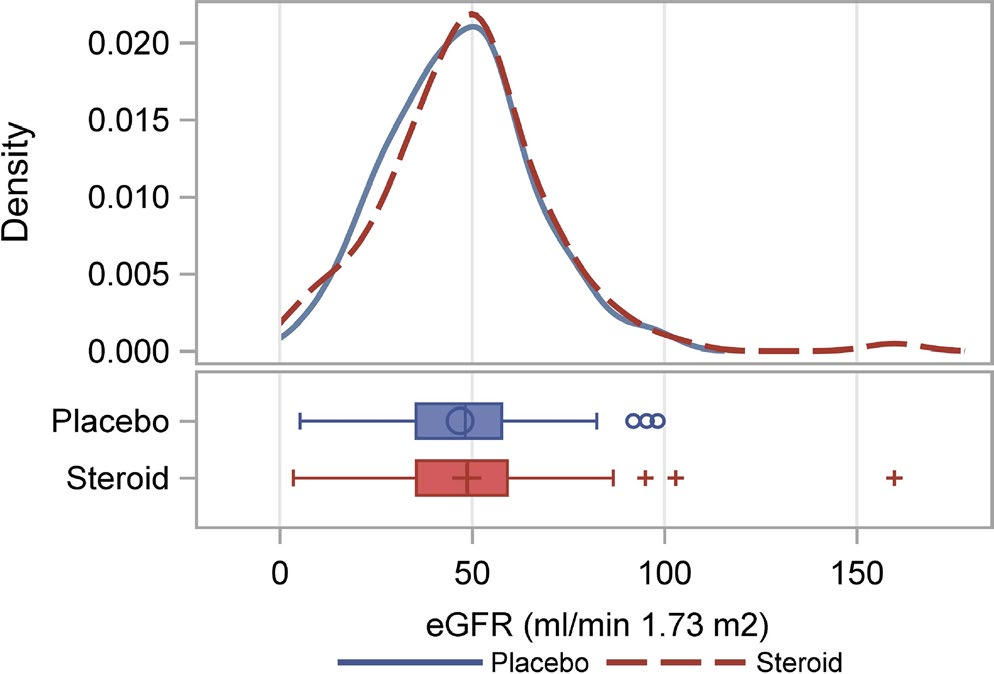
Distribution of eGFR at 5 years after transplantation. The patient with an eGFR >150 mL/min was a 14‐year‐old child who received an adult donor kidney. eGFR, estimated glomerular filtration rate

Change in kidney allograft function estimated by eGFR trajectories over 5 years showed no difference between the 2 groups (mean eGFR difference ‐0.25 mL/min per 1.73 m^2^, 95% CI −3.21 to 3.71, *P* = .887) (Figure 3, Table S2). The distribution of eGFR values at 5 years was 48 mL/min per 1.73 m^2^ in the steroid group and 47 mL/min per 1.73 m^2^ in the placebo group, respectively (Figure 4). The 1 outlier was a 14‐year‐old child who received an adult donor kidney.

## 4 DISCUSSION

Here, we report the long‐term outcome of our randomized controlled trial on systemic anti‐inflammatory treatment of deceased organ donors using high doses of corticosteroids prior to organ retrieval. A single dose of 1000 mg methylprednisolone infused to the donor at least 3 hours prior to organ recovery did not reduce early BCAR and improve long‐term graft survival at 5‐year follow‐up.

We previously reported the short‐term outcomes of the trial and found no difference in the rate of delayed graft function between steroid pretreatment and placebo group.^12^, ^16^ However, single dose of steroid infusion resulted in a reduction of inflammatory signals in gene expression analysis of preimplantation biopsies, suggesting adequate timing and dosing of the intervention.

Brain death causes hemodynamic instability, induces hormonal changes, and results in a cascade of inflammatory events and endothelial cell activation.^17^ Inflammation and endothelial cell activation increases HLA expression on endothelial cells.^18^ We therefore hypothesized that anti‐inflammatory treatment may impact on the rate of acute rejection episodes within the early posttransplant period.

Despite the reduction of inflammation in the preimplantation biopsies, there was no statistically significant difference detectable in the incidence of acute rejection between both groups. There was no episode of antibody‐mediated rejection observed within the first 3 months, which may be explained by the overall low immunological risk of the study population. Eighty‐seven percent of patients had received their first transplant and exhibited an overall very low level of sensitization. Only 38 patients (8.6%) had panel‐reactive antibody levels >10% at time of transplantation. Donor‐specific antibodies were not tested routinely at the time of study enrollment.

Donor studies can only be initiated after declaration of brain death. This limits the window for intervention to only a few hours before organ explant. Given this short time interval, sequential steroid doses were not feasible in this setting. In our study, the single steroid bolus was administered at least 3 hours before organ procurement. No additional steroid treatment was performed following declaration of brain death in the donors. However, we do not have information on treatment of donors prior to inclusion in the study.

Several other strategies to reduce the rate of acute injury and delayed graft function and improve long‐term outcome following donation after brain death have been assessed. This includes optimized care of the deceased brain‐dead donor such as dopamine pretreatment, hypothermia of donors prior to organ explantation, as well as ex vivo machine perfusion of the organs.^19^, ^20^, ^21^, ^22^, ^23^ Only a few interventions have been shown to ameliorate ischemia‐reperfusion injury in the allograft. This includes permissive hypothermia of the donor and ex vivo machine perfusion of the explanted organs.^19^, ^20^ However, these strategies are currently not routinely applied in most transplant programs, and their impact on early BCAR and long‐term graft survival remains elusive.

Most interventional trials on donor pretreatment focused on the early postoperative period using delayed graft function as primary endpoint.^19^, ^21^, ^24^ Schnuelle et al recently reported the long‐term follow‐up of dopamine pretreatment of donors. In the intention‐to‐treat analysis, no survival advantage was observed. Subgroup analysis showed a nonlinear time‐dependent effect suggesting a potential survival benefit in donors with longer treatment duration. Currently a study on cyclosporine pretreatment of donors is enrolling patients.^25^ However, our previous study showed that downregulation of inflammation signatures in preimplantation biopsies after steroid treatment did not impact on posttransplant outcomes.

A recent systematic review concluded that there was not enough evidence to draw definite conclusions on the potential benefits of steroid pretreatment of organ donors.^11^ The need for high‐quality data on long‐term outcome following steroid treatment of donors has been argued in another review.^23^ Our data emphasize that despite significant reduction of inflammation in the allograft, steroid pretreatment of donors did not improve long‐term graft function. Five‐year graft survival in both groups was excellent and comparable to recent trials of first transplant recipients and standard criteria donors.^26^, ^27^

Our present study exhibits some limitations. The study was powered to detect a robust difference in the HR for graft loss but therefore a modest difference might have been missed. At time of patient enrollment, no standardized testing for donor‐specific HLA antibodies was performed. The observed delayed graft function (DGF) rates in the trial were 22% and 25% in the steroid and placebo group, respectively.^12^ This is in line with previous reports from the Eurotransplant region that showed a DGF rate ≈23% for the period from 1990 to 2003 on which power analysis was based.^28^ The rate of extended criteria donors was 20.1% in our cohort and thus representative for both US and European deceased donor transplant programs.^29^ We had no data on kidney donor risk index (KDPI). However, in a donor population of higher DGF risk including donation after circulatory death and high KDPI, repetitive and higher doses of steroid treatment may result in different findings. Interaction analysis did not show a significant effect modification of steroid pretreatment on graft survival for donors older than 50 years or with higher creatinine levels (Figure 2 and Table S3).

The strength of this analysis is the blinded randomized controlled study design and the high rate of complete follow‐up in >97% of enrolled patients, allowing for a high internal validity. The multicenter study design ensures high external legitimacy of our findings and is thus representative for donor and recipient characteristics in Europe and likely also in the United States, although the study population was mainly of white ethnicity.

In summary, our data show that systemic anti‐inflammatory treatment of deceased organ donors using high‐dose steroids prior to organ retrieval did not reduce early BCAR and did not improve long‐term graft survival.

## DISCLOSURE

The authors of this manuscript have no conflicts of interest to disclose as described by the *American Journal of Transplantation*.
