# Supplementary material for: Steroid pretreatment of organ donors does not impact on early rejection and long‐term kidney allograft survival: Results from a multicenter randomized, controlled trial
Source: Am J Transplant. 2019 Feb 1;19(6):1770–6. doi: 10.1111/ajt.15252 (PMC6563104; doi:10.1111/ajt.15252)

Supplement to:

Steroid pretreatment of organ donors does not impact on early rejection and long-term kidney allograft survival – results from a multicenter randomized, controlled trial

Roman Reindl-Schwaighofer, MD^1^*, Alexander Kainz PhD^1^*, Kira Jelencsics, PhD^1^, Andreas Heinzel, MS^1^, Gabriela Berlakovich, MD^2^, Ádám Remport, MD^3^, Georg Heinze, PhD^4^, Robert Langer, MD^5^, and Rainer Oberbauer, MD^1^

1 Department of Nephrology, Medical University of Vienna, Austria

2 Department of Surgery, Medical University of Vienna, Austria

3 Department of Nephrology, Semmelweis University, Budapest, Hungary

4 Center for Medical Statistics, Informatics and Intelligent Systems (CEMSIIS), Medical University of Vienna, Austria

5 Department of Surgery, Elisabethinen Krankenhaus, Linz, Austrianen Krankenhaus, Linz, Austria

* Contributed equally

Table S1. BANFF Score of BCAR according to treatment group

|  | Steroid treatment (n=228) | Placebo (n=212) |
| --- | --- | --- |
| BANFF 1 | 18 (7.9%) | 20 (9.4%) |
| BANFF 2 | 5 (2.2%) | 6 (2.8%) |

Table S2. Parameter estimates for mixed model of eGFR slopes per years. Intercept and year were also included as random variables.

| Effect | Estimate | 95% confidence Limits | | p-value |
| --- | --- | --- | --- | --- |
| Intercept | 45.17 | 42.68 | 47.66 | <0.001 |
| Treatment (steroid vs placebo) (ml/min 1.73m2) | 0.25 | -3.21 | 3.71 | 0.887 |
| Year (ml/min 1.73m2 year) | 0.16 | -0.93 | 1.26 | 0.771 |
| Interaction year, treatment (ml/min 1.73 m2 year) | 0.33 | -1.19 | 1.85 | 0.669 |

Table S3: Interaction analysis of treatment assignment (steroid vs placebo stratified by donor age and donor creatinine levles)

|  | Hazard ratio | 95% confidence Limits | | p-value for interaction |
| --- | --- | --- | --- | --- |
| **Donor age ≤ 60** (n=349) **/ > 60** (n=91) | | | | |
| Donor age ≤ 60 | 0.95 | 0.55 | 1.65 | 0.548 |
| Donor age > 60 | 0.70 | 0.31 | 1.59 |  |
| **Donor age ≤ 65** (n=388) **/ > 65** (n=52) | | | | |
| Donor age ≤ 65 | 1.00 | 0.60 | 1.69 | 0.404 |
| Donor age > 65 | 0.61 | 0.21 | 1.75 |  |
| **Donor age ≤ 70** (n= 409) **/ > 70** (n=31) | | | | |
| Donor age ≤ 70 | 0.98 | 0.60 | 1.745 | 0.247 |
| Donor age > 70 | 0.38 | 0.08 | 1.75 |  |
| **Donor creatinine ≤ 1.5 mg/dl** (n= 413) **/ > 1.5 mg/dl** (n=27) | | | | |
| Donor age ≤ 1.5 mg/dl | 0.89 | 0.55 | 1.45 | 0.890 |
| Donor age > 1.5 mg/dl | 0.98 | 0.29 | 3.26 |  |

Figure S1

Flowchart according to CONSORT.


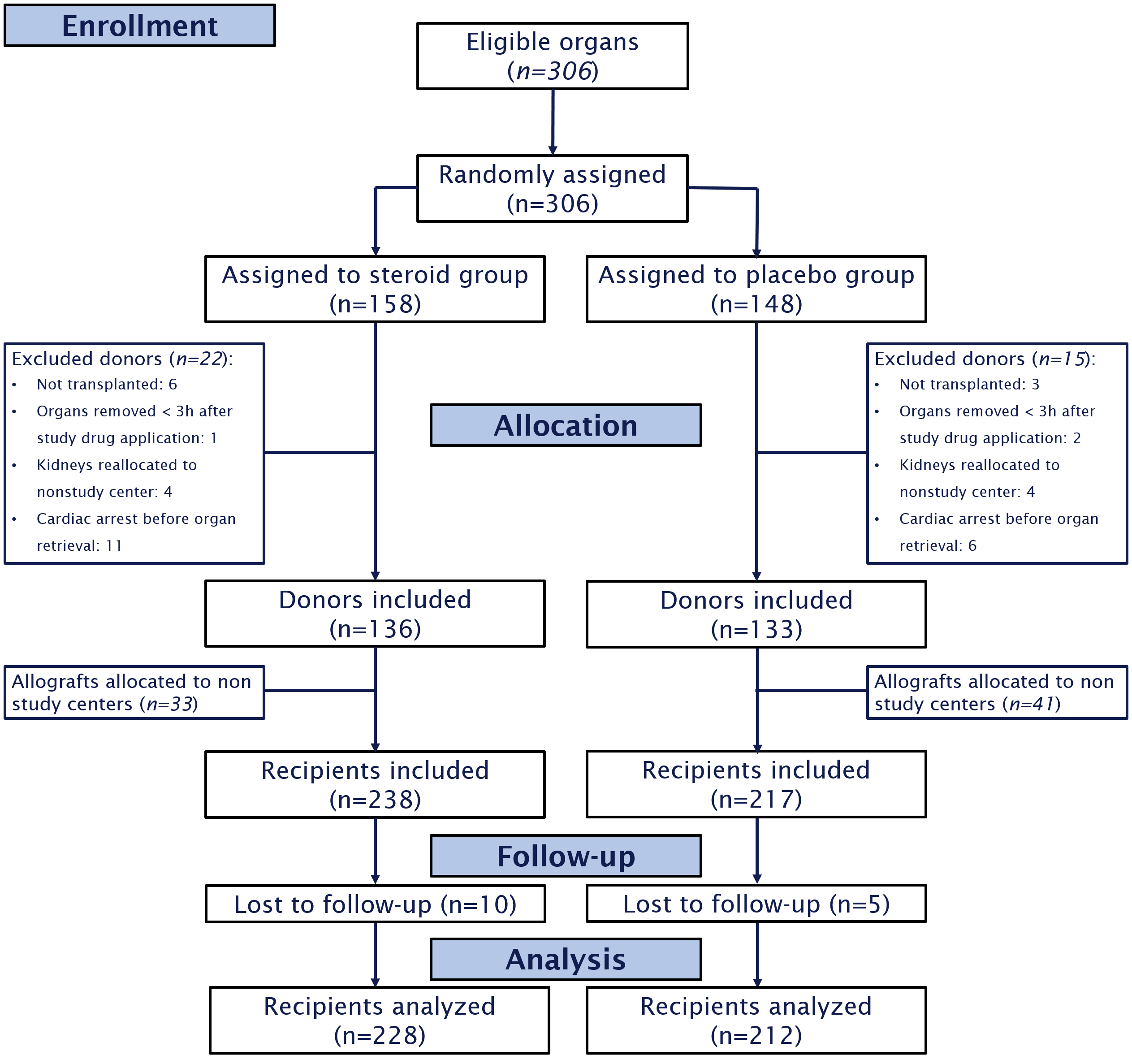


Figure S2

Detectable hazard ratio for the available data set.

The detectable effect size was calculated with the following parameters:

α = 0.05

number of patients per group = 220

median survival time = 10 years

accrual time = 0 years (all patients are already included)

follow up time = 5 years


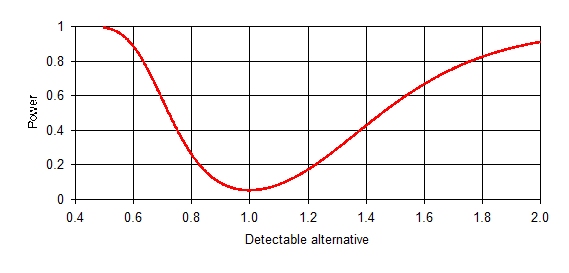


Figure S3

Actual graft survival (graft loss and death as events). Numbers on x-axis indicate the patients at risk.


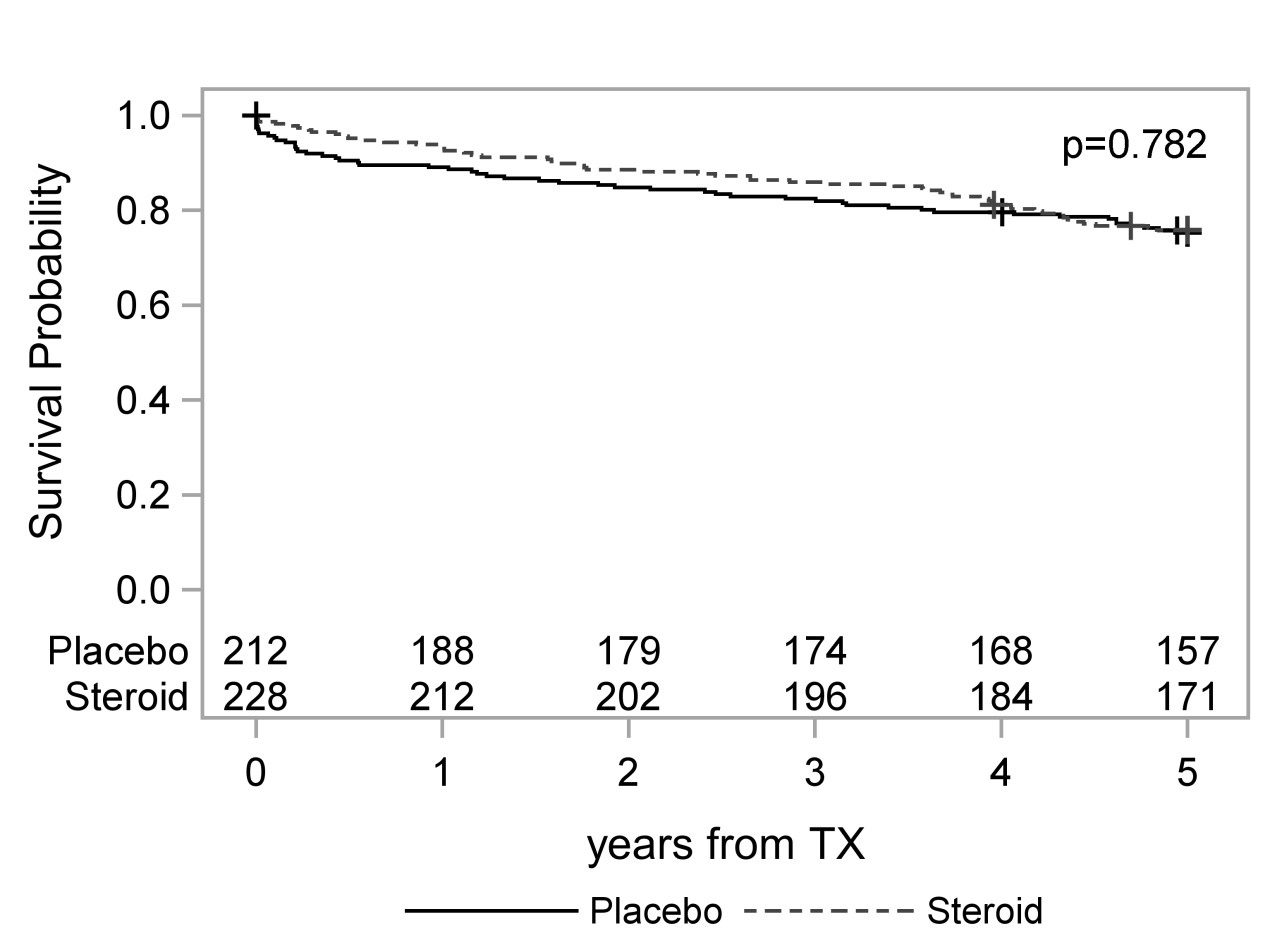


Figure S4

Patient survival. Numbers on x-axis indicate the patients at risk.


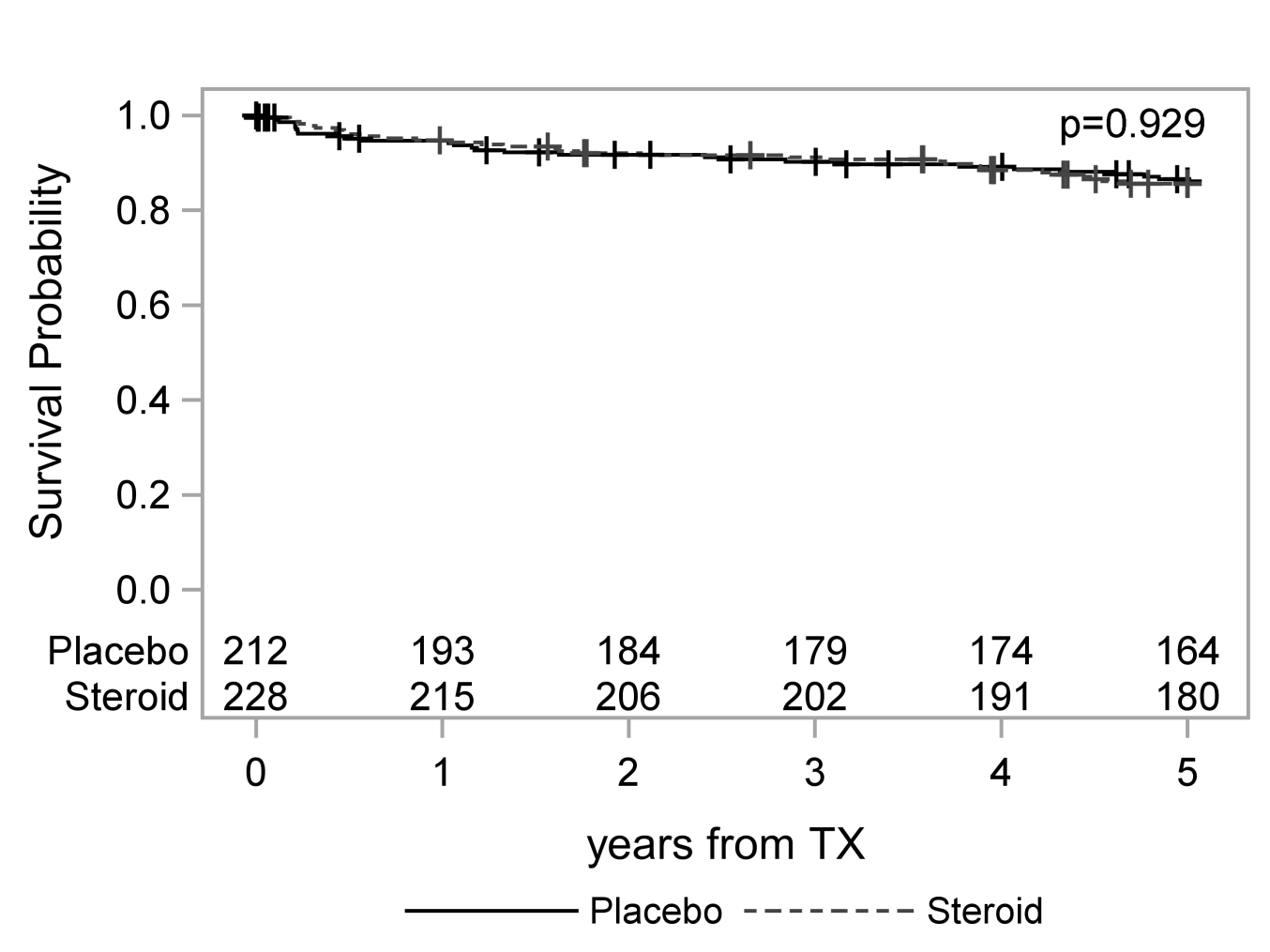


Figure S5

Cumulative incidence for functional graft loss. Death was counted as a competing event.


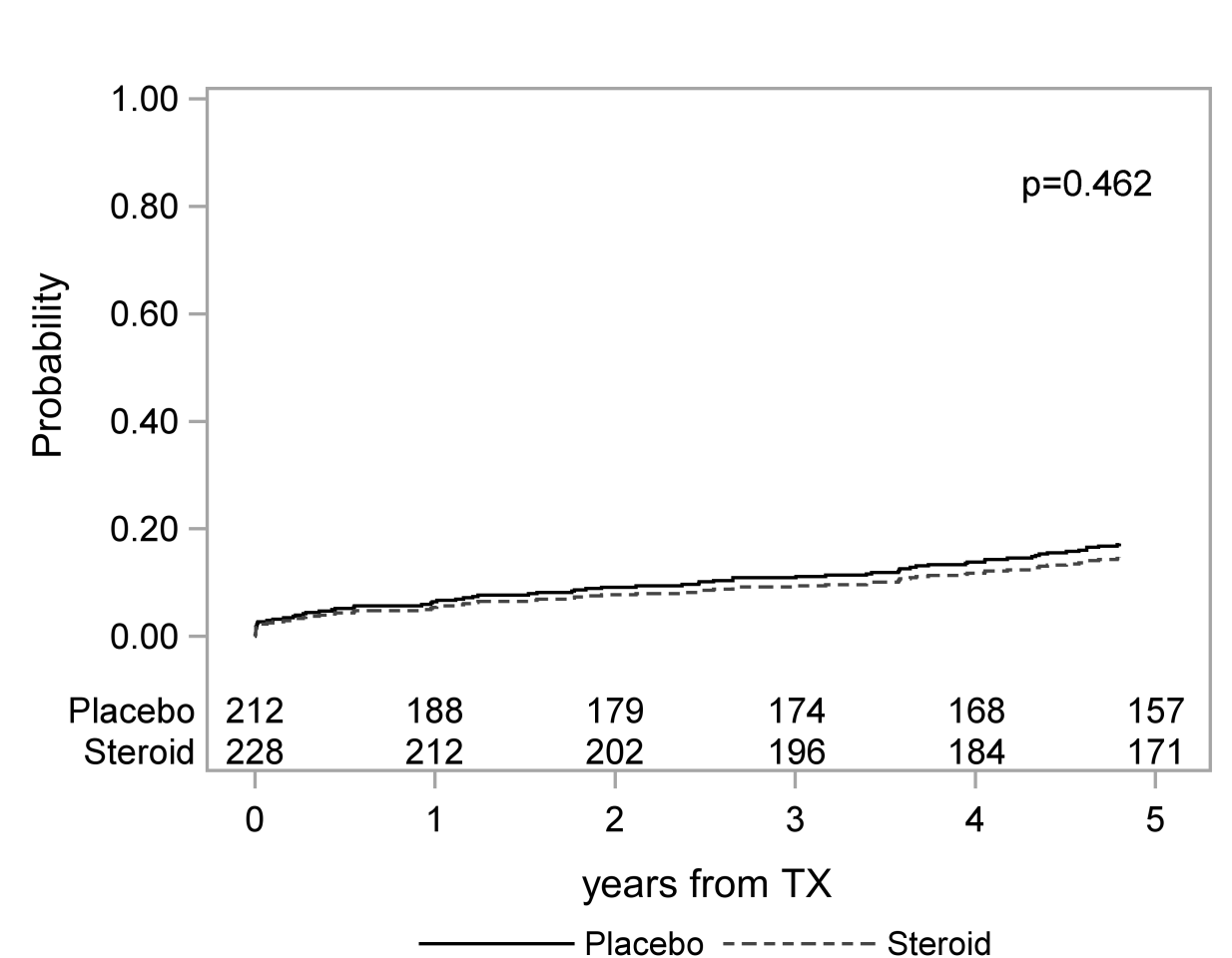

Supplement: Supplementary file 1 [file AJT-19-1770-s001.docx]
